# Prevalence of Comorbidity and Associated Factors Among Patients With Hypertension in Health Facilities of Southern Ethiopia

**DOI:** 10.1155/bmri/7739774

**Published:** 2026-01-21

**Authors:** Habtamu Endashaw Hareru, Banchayehu Azmeraw Gedamu, Mequanint Ayehu Akele, Endashaw Kefyalew Temesgen, Daniel Sisay

**Affiliations:** ^1^ School of Public Health, College of Health Sciences and Medicine, Dilla University, Dilla, Ethiopia, du.edu.et; ^2^ Wonago Health and Demographic Surveillance System, Dilla University, Wonago, Ethiopia, du.edu.et; ^3^ School of Medicine, College of Health Sciences and Medicine, Dilla University, Dilla, Ethiopia, du.edu.et; ^4^ Department of Nursing, College of Health Sciences and Medicine, Dilla University, Dilla, Ethiopia, du.edu.et

**Keywords:** associated factors, comorbidity, Ethiopia, high blood pressure, noncommunicable diseases, patients with hypertension

## Abstract

**Background:**

Comorbidity in patients with hypertension presents a significant challenge to effective disease management, leading to worsened health outcomes due to increased complications, higher healthcare costs, and elevated mortality rates. In resource‐limited settings like Ethiopia, the prevalence of comorbid disorders among patients with hypertension remains poorly understood. This lack of understanding is particularly alarming as noncommunicable diseases are on the rise, and the health systems in these areas are often inadequate. While different studies have assessed the prevalence and risk factors of hypertension, there is limited knowledge regarding the prevalence of comorbidity and related factors among patients with hypertension, particularly in Southern Ethiopia. To effectively develop integrated methods for the prevention, early diagnosis, and management of hypertension, a thorough understanding of the associated comorbidities and related factors is essential. Therefore, this study is aimed at determining the prevalence of comorbidity and its associated factors among patients with hypertension in healthcare facilities of Southern Ethiopia.

**Methods:**

An institution‐based cross‐sectional study was conducted among patients with hypertension aged ≥ 18 years attending follow‐up at Dilla University Teaching Hospital from November 30, 2022, to January 30, 2023. A systematic random sampling technique was used to recruit 190 hypertensive patients from a hypertension registration logbook, collected using a pretested, structured‐administered questionnaire and supplemented by a medical record review. The data collection tool was translated into the Amharic language and back‐translated to ensure consistency. The data was analyzed using STATA Version 14. To characterize the data, descriptive statistics were applied. To determine the factors that are associated with comorbidity among patients with hypertension, a logistic regression model was fitted. Model fitness was checked using the Hosmer–Lemeshow goodness‐of‐fit test (*p*‐value = 0.633), indicating an adequate model fit. In the end, an adjusted odds ratio (AOR) with a 95% confidence interval (CI) was estimated and interpreted, and a *p*‐value of less than 0.05 was used to declare statistical significance.

**Result:**

The prevalence of comorbidity among patients with hypertension was 56.8% (95% CI: 49.7%–63.7%). Several factors were found to be significantly associated with comorbidity. Patients aged 55 years and above were over four times more likely to have comorbid conditions (AOR = 4.42, 95% CI: 2.65–7.68), while those residing in urban areas had nearly seven times higher odds of comorbidity (AOR = 6.67, 95% CI: 2.73–8.72). Likewise, alcohol consumption (AOR = 3.50, 95% CI: 2.94–11.31), cigarette smoking (AOR = 4.87, 95% CI: 1.23–6.53), and having a body mass index (BMI) ≥ 25 kg/m^2^ (AOR = 4.55, 95% CI: 2.78–10.91) were independently associated with a higher likelihood of comorbidity. Conversely, being physically active was inversely associated with comorbidity, reducing the odds by nearly half (AOR = 0.52, 95% CI: 0.24–0.89).

**Conclusion:**

More than half of the patients with hypertension had at least one comorbid condition. Sociodemographic, behavioral, and clinical factors were significantly associated with these comorbidities. Based on our findings, we recommend routine screening for comorbid conditions, the implementation of educational programs to increase patient awareness about the risks of alcohol consumption and smoking, the promotion of healthy lifestyle practices, and improved access to healthcare services to ensure early detection and comprehensive management of hypertension and its related comorbidities.

## 1. Introduction

Noncommunicable diseases (NCDs) are a major global public health concern, accounting for 71% of all deaths worldwide [1] and more than half of the global burden of disease [2]. According to the 2019 World Health Organization (WHO) report, NCDs have the greatest impact in low‐ and middle‐income countries (LMICs), where they account for 78% of all deaths, 85% of premature mortality, and 50% of disabilities [1, 3]. Hypertension is one of the most prevalent NCDs in both developed and developing countries, including Ethiopia [4]. It is defined as a persistent systolic blood pressure (SBP) of 130 mmHg or higher, a diastolic blood pressure (DBP) of 90 mmHg or higher, or the current use of antihypertensive medications [5]. Globally, hypertension affects approximately 50% of individuals aged 60 years and above [6]. It remains a major public health threat, affecting an estimated 1.39 billion people worldwide and overcontributing to more than 10.8 million deaths annually [7]. Hypertension is also the leading cause of mortality related to stroke and coronary heart disease [8]. In its global action plan for the prevention and control of NCDs, the WHO projected a 25% reduction in the prevalence of hypertension between 2010 and 2025 [9].

The rising prevalence of hypertension is influenced by several factors, including high body mass index (BMI), alcohol consumption, tobacco use, emotional stress, and unhealthy dietary and lifestyle habits [10–12]. Its public health impact is further compounded by frequent coexistence with other NCDs, which collectively lead to a reduced quality of life, diminished work capacity, increased healthcare costs, and greater strain on health systems [12, 13] Among hypertensive patients, the most common comorbid conditions include diabetes mellitus, chronic kidney disease, hyperlipidemia, and cardiovascular disorders [3, 10]. Managing these multiple comorbidities presents significant challenges for both patients and healthcare providers [14]. Previous studies have emphasized the need for greater attention to comorbidities during the clinical assessment and treatment planning, as prioritizing their management can substantially improve disease control and overall patient outcomes [10, 15].

Studies conducted across different countries showed considerable variation in the prevalence of comorbidities among patients with hypertension, ranging from 23% in the United States to 100% in India [13, 16–18]. The variation could be attributed to differences in the reference population, inclusion of the disease categories, the operational definition of comorbidity, study design, and data representativeness. Some studies showed that the presence of comorbidities in patients with hypertension is influenced by various sociodemographic, clinical, and behavioral factors. Key contributors include older age, male gender, obesity, a longer duration of hypertension, and inadequate treatment [17, 19–21], as well as lifestyle factors such as physical inactivity, excessive salt intake, and substance use (e.g., alcohol and smoking). Together, these factors increase the likelihood of developing comorbid conditions in individuals with hypertension [17, 22].

In Ethiopia, several studies have examined the prevalence of hypertension and its associated factors [23–28]. A recent systematic review and meta‐analysis study revealed that the pooled prevalence of hypertension, undiagnosed hypertension, and uncontrolled hypertension in Ethiopia was 28.02% [29], 18.26% [30], and 51% [31], respectively. In addition, a few studies have assessed specific comorbid conditions among patients with hypertension. These studies found that the prevalence of chronic kidney disease among individuals with hypertension ranged from 17.6% to 22.1% [32, 33], the comorbidity of Type 2 diabetes mellitus ranged from 18.8% to 59.7% [12, 33], and approximately 90.8% of hypertensive patients had at least one dyslipidemia [34]. However, there is limited evidence regarding the overall prevalence of comorbidity and its associated factors among hypertensive patients in the study area.

Therefore, this study was aimed at determining the prevalence of comorbidity and associated factors among patients with hypertension. This study could help hospitals and healthcare planners develop integrated prevention programs, early detection of comorbid diseases, and effective management of comorbidity, in addition to the primary disease (hypertension) to achieve better treatment outcomes. It is also used to inform the hospital and facilitate the establishment of multidisciplinary treatment approaches by bringing together several specialties in one location to allocate public health resources effectively.

## 2. Materials and Methods

### 2.1. Study Setting and Period

The study was conducted at Dilla University Teaching Hospital, which is located in the capital of Gedeo Zone, Dilla City Administration, Southern Ethiopia, at a distance of 365 km from the capital city of Ethiopia, Addis Ababa. The hospital is a key referral center offering 24‐h emergency services and comprehensive care to over 5 million people, mainly from the Gedeo Zone and surrounding regions. It employs over 1500 staff, including more than 60 specialists. The hospital provides the following services: internal medicine, surgery, obstetrics, pediatrics, dentistry, ophthalmology, radiology, pharmacy and clinical laboratory, nursing, and midwifery services. Moreover, the hospitals establish the use of electronic health records, perform various problem‐solving research, offer patient education programs supervised by healthcare professionals, and participate in community outreach activities. Moreover, as a teaching hospital, the hospital gives medical education to a variety of categories of health professionals. This study was conducted from November 30, 2022, to January 30, 2023.

### 2.2. Study Design

An institution‐based cross‐sectional study design was conducted to determine the comorbidities and associated factors among patients with hypertension attending their follow‐up at Dilla University Teaching Hospital, Dilla, Southern Ethiopia.

### 2.3. Population

All hypertension patients under follow‐up at Dilla University Teaching Hospital were the source population. All patients with hypertension who were available during the data collection period were the study population. Patients with hypertension aged 18 and above who had been on follow‐up for at least 6 months were included in the study. Adults with severe medical illnesses and cognitive impairments (unable to hear), such as patients with severe mental disabilities who were unable to participate and pregnant women who needed specific hypertension care, were excluded.

### 2.4. Study Variables

The dependent variable was the presence of comorbidity among patients with hypertension. The independent variables included sociodemographic factors (such as age, sex, educational level, occupation, monthly income, marital status, and place of residence), behavioral factors (including cigarette smoking, alcohol consumption, and physical activity), and clinical factors (such as family history of hypertension, duration of hypertension, and BMI).

### 2.5. Sample Size Determination

The sample size (*n*_*i*_) was determined using a single population proportion formula, taking into account the prevalence of comorbidity among hypertensive patients from a previous study in Northwest Ethiopia, which was 21.1% [33], a two‐sided 95% confidence level (*Z*_*α*/2_ = 1.96), and a 5% margin of error. *n*_*i*_ = [(*Z*(*a*/2))^2^ *p*(1 − *p*)]/*d* ^2^ = [(1.96)^2^0.21(1 − 0.21)]/(0.05)^2^ ≈ 255. Since the entire population is less than 10,000, we applied the population correction formula: nf = *n*_*i*_/(1 + *n*_*i*_/*N*) ; nf = nf = 255/(1 + 255/693) = 186. As a result, assuming a 10% nonresponse rate, the optimal sample size for the current study was 205.

### 2.6. Sampling Technique and Procedure

The 2‐month report revealed that 693 hypertensive patients at Dilla University Teaching Hospital were under chronic follow‐up. To select individual samples, a systematic random sampling method was employed. The sampling interval (*k*) was calculated by dividing the total population size by the desired sample size (*k* = *N*/nf = 693/205 = 3.3 ≈ 3), where *N* represents the total number of hypertension patients available during the data collection period and nf denotes the sample size. Using the lottery method, a random number between 1 and 3 was chosen to identify the first participant. Subsequently, participants were selected at every third interval using the systematic random sampling technique until the required sample size was achieved.

### 2.7. Operational Definitions

#### 2.7.1. Hypertension

Hypertension was defined as having a blood pressure of ≥ 130/90 mmHg, taking antihypertensive medication, being diagnosed by a healthcare professional, having it documented in medical records, or self‐reported by participants [35].

#### 2.7.2. Comorbidity

To assess the comorbidity status of patients with hypertension, we used self‐reported data from the participants and a review of their medical records. The participants were asked the following question: “Have you ever been diagnosed by a healthcare professional with any of the following chronic conditions (i.e., diabetes mellitus, heart failure, dyslipidemia, stroke, and chronic renal disease)?” Participants′ responses were recorded as binary values (1 for “yes” response and 0 for “no” response). To minimize recall bias, self‐reported information on comorbid conditions was cross‐verified through medical record review. Participants with one or more additional chronic diseases were categorized as having comorbidity in a patient diagnosed with hypertension. Moreover, patients with multiple chronic diseases were still categorized under “comorbidity” (one or more conditions).

#### 2.7.3. Current Smoker

To assess smoking status, participants were asked: “How many of the past 28 days did you smoke a cigarette or cigar, even just one puff?” Respondents who reported “no” smoking for 0 days were considered nonsmokers; otherwise, they were classified as smokers.

#### 2.7.4. Physical Activity

It was defined as engaging in vigorous exercise for at least 3 days per week for 30 min or longer. Moderate exercise is defined as engaging in vigorous exercise for 3 days per week, each lasting 20 min, or 5 days of walking, swimming, or biking for at least 30 min each day. Low activity is defined as any activity that falls short of the categories listed above [36]. In this study, individuals engaging in moderate to high levels of physical activity were categorized as physically active, while those with low or no physical activity were categorized as inactive. The physically active group was labeled as “yes,” and the inactive group was labeled as “no.”

#### 2.7.5. Current Alcohol Drinkers

Participants who reported consuming manufactured alcoholic beverages (i.e., a bottle of regular beer or other drinks [300 mL] or one medium‐sized glass of wine [120 mL] of alcohol) or any local beverage (i.e., *areki*, *tella*, *tej*, and *katikalla*) in the past week were classified as “alcohol drinkers,” while those who reported not drinking any alcohol in the past week were considered “nondrinkers” [37].

#### 2.7.6. Family History of Hypertension

It was categorized as “yes” if patients with hypertension had a history of hypertension in his/her father, mother, or full brother or sister; otherwise, “no” for family history of hypertension.

### 2.8. Data Collection Techniques and Procedures

Data were collected by using a pretested, structured, interviewer‐administered questionnaire, and the patient′s medical records were also reviewed to gather further information from the history, physical examination, and investigations. The data collection tools comprised sociodemographic variables, behavioral variables, and clinical variables, which were adapted from reviewing different literatures [10–12, 16, 35]. The data collection tools were translated to the local language by a local language expert and then back‐translated to English to maintain consistency. The study participants′ weight and height were measured to determine their BMI.

The participants′ BMI (BMI = weight in kg/height in m^2^) was calculated and classified as underweight (< 18.5), normal weight (18.5–24.9), overweight (25–29.9), or obese (≥ 30) [38]. Moreover, the medical histories of the participants (i.e., dyslipidemia, cardiovascular disease, stroke, heart failure, diabetes mellitus, and kidney disease) were gathered from the patients′ medical records and registration books. The questionnaires were initially prepared in XLSX format using a Microsoft Excel sheet. They were then converted to X‐form using an online XLSX converter for data collection via KoboToolbox. The KoboToolbox application (Version 2022.4.4) was installed on the data collector′s Android mobile phone to facilitate this process. The data were collected by four clinical nurses working in the hospital and supervised by one health officer who had experience in research activities.

### 2.9. Data Quality Control

To ensure data quality, we utilized standardized tools in KoboCollect to minimize variability in responses. This involved providing clear instructions and predefined response options. Additionally, we implemented validation rules within KoboCollect to restrict entries to acceptable values, such as numeric ranges for age and predefined lists for comorbidities. These measures help reduce the likelihood of erroneous data entry. The questionnaire was translated into the local language (Amharic) and back‐translated to English by language experts to maintain consistency. Data collectors and supervisors underwent 2 days of training focused on the purpose and significance of the study, ethical considerations, data collection techniques and procedures, how to use KoboToolbox for data collection on Android mobile phones, and the importance of accurate data entry. The data collection tools were pretested on 5% of the computed sample size at another hospital for relevance, completeness, and clarity in answering the study question, and modifications were made as needed prior to the actual data collection period. Each day, we reviewed the questionnaire to ensure its completeness and accuracy before the data collectors uploaded the gathered information to the KoboToolbox server from their devices. The principal researcher and supervisor carefully oversaw the overall process of data collection.

### 2.10. Data Processing and Analysis

After collecting data with KoboToolbox, it was downloaded as a Microsoft Excel file and exported to STATA Version 14.0. The data was then checked for completeness, consistency, missing values, and outliers and then cleaned, coded, and subjected to further statistical analysis. Descriptive statistics were utilized to compute and present the study participants′ sociodemographic, behavioral, and clinical characteristics. For categorical variables, frequencies and percentages were used. For continuous variables, the mean and standard deviation (SD) were reported. A binary logistic regression model was used to determine the relationship between independent variables and comorbidity among hypertensive patients. Variables associated with comorbidity (at a *p*‐value less than 0.25) in bivariable logistic regression were incorporated into the multivariable logistic regression model in order to account for confounders. Multicollinearity was assessed using the variance inflation factor (VIF) for continuous variables and contingency coefficient values for categorical variables. No significant multicollinearity was detected (VIF < 10 for continuous variables and contingency coefficient < 0.7 for categorical variables). Hence, all variables were retained in the final model. Hosmer–Lemeshow tests determined a model′s goodness of fit, with a *p*‐value > 0.05 indicating a well‐fit model. As a result, our study′s model was a good fit (*p*‐value = 0.633). Finally, the factors with significant associations were selected by calculating the odds ratio with a 95% confidence interval, and a *p*‐value less than 0.05 was used to declare the statistical significance in the multivariable logistic regression model. The data was organized and presented using text, tables, and figures.

### 2.11. Ethical Consideration

Ethical approval for the study was granted by the Institutional Review Board of Dilla University, College of Health Sciences and Medicine, under Approval Number duirb/053/22‐10.

A cooperation letter was obtained from the School of Medicine, and verbal informed consent was secured from each participant after providing a clear explanation of the study′s methodology, objectives, and purpose. Privacy and confidentiality were strictly maintained, with specific measures implemented for participants facing literacy challenges or logistical barriers to written consent. The data collector recorded the verbal consent in the study log, including details such as the date, time, and participant number to ensure accountability and transparency.

## 3. Result

### 3.1. Sociodemographic Characteristics of Study Participants

The study included 190 patients diagnosed with hypertension, yielding an overall response rate of 92.7%. The participants had an average age of 59.42 years, with a SD of 13.81 years. Of the total participants, 97 (51.05%) were male. About 25.2% had completed secondary education, and over one‐third (38.42%) were employed.

Furthermore, 50.53% of the participants lived in urban areas. The average monthly income was 2101 Ethiopian birr (ETB), with a SD of 1104 ETB. Approximately one‐third (33.16%) reported a monthly income ranging between 1000 and 3000 ETB (Table 1).

**Table 1:** Sociodemographic characteristics of patients with hypertension admitted at Dilla University Teaching Hospital, Southern Ethiopia, Nov 30, 2022, to Jan 30, 2023 (*n* = 190).

**Variables**	**Frequency**	**Percentage (%)**
Sex		
Male	97	51.05
Female	93	48.95
Age		
18–34	26	13.69
35–54	86	45.26
≥ 55	78	41.05
Marital status		
Single	36	18.95
Married	97	51.05
Divorced	13	6.84
Widowed	44	23.16
Place of residence		
Urban	96	50.53
Rural	94	49.47
Educational status		
Unable to read and write	56	29.48
Grades 1–8 (primary)	67	35.26
Grades 9–12 (secondary)	48	25.26
College and above	19	10.00
Occupational status		
Unemployed	41	21.58
Employed	73	38.42
Farmer	40	21.05
Merchant	36	18.95
Monthly income (ETB) of the participants		
< 1000	77	40.52
1000–3000	63	33.16
> 3000	50	26.32

Abbreviation: ETB, Ethiopian birr.

### 3.2. Behavioral Characteristics

Out of 190 hypertension patients, the majority of the participants (80.53%) had never smoked, 74.74% did not consume alcohol, and 78.42% were physically inactive (Table 2).

**Table 2:** Behavioral characteristics of patients with hypertension admitted at Dilla University Teaching Hospital, Southern Ethiopia, Nov 30, 2022, to Jan 30, 2023 (*n* = 190).

**Variables**	**Frequency**	**Percentage (%)**
Cigarette smoking		
Yes	37	19.47
No	153	80.53
Alcohol drinking		
Yes	48	25.26
No	142	74.74
Physical activity		
Yes	41	21.58
No	149	78.42

### 3.3. Clinical Characteristics

The average BMI (±SD) of the participants in this study was 23 (±2) kg/m^2^, with nearly two‐thirds classified within the normal BMI range (18.5–24.9). Among the 190 patients with hypertension, over half (54.74%) reported no family history of the condition, and a majority (53.2%) had been experiencing hypertension for 1–5 years (Table 3).

**Table 3:** Clinical characteristics of patients with hypertension admitted at Dilla University Teaching Hospital, Southern Ethiopia, November 30, 2022, to January 30, 2023 (*n* = 190).

**Variables**	**Frequency**	**Percentage (%)**
Family history of hypertension		
Yes	86	45.26
No	104	54.74
BMI		
Underweight (BMI < 18.5)	37	19.47
Normal range (18.5–24.9)	122	64.21
Overweight or obese (BMI ≥ 25)	31	16.32
Duration with hypertension (in years)		
1–5	101	53.16
6–10	69	36.31
≥ 11	20	10.53

### 3.4. Prevalence of Comorbidities Among Patients With Hypertension

**Figure 1:**
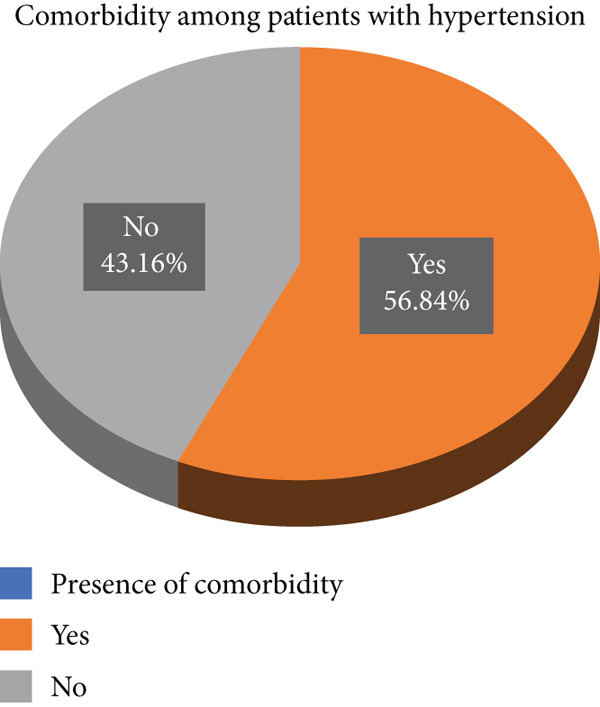
Prevalence of comorbidities among patients with hypertension admitted at Dilla University Teaching Hospital, Southern Ethiopia, Nov 30, 2022, to Jan 30, 2023 (*n* = 190).

Among the 190 hypertensive patients, 108 (56.84%; 95% CI: 49.73%, 63.68%) had at least one comorbid condition (Figure 1). Out of 190 patients with hypertension, 52 (27.37%) had diabetes mellitus, while 43 (22.63%) were diagnosed with heart failure. Additionally, dyslipidemia was observed in 38 patients (20%), stroke in 17 patients (8.95%), and chronic renal disease in 16 patients (8.42%). The sum of patients with each listed comorbidity is much higher than the total number of patients with a comorbidity. That is, diabetes (52), heart failure (43), dyslipidemia (38), stroke (17), and chronic renal disease (16) equal 166 patients. Some patients had more than one chronic disease (multimorbidity); however, for this study, the presence of one or more chronic conditions was classified as comorbidity. Therefore, the sum of individual comorbid diseases exceeds the total number of patients with comorbidity (108).

### 3.5. Factors Associated With Comorbidities Among Patients With Hypertension

In the bivariate logistic regression analysis, variables such as gender, age, occupational status, residence, monthly income, alcohol consumption, physical activity, duration of hypertension, cigarette smoking, and BMI were considered for inclusion in the multivariable logistic regression analysis, using a *p*‐value of less than 0.25. In the subsequent multivariable logistic regression analysis, with a *p*‐value of less than 0.05, age 55 or older, urban residence, alcohol consumption, cigarette smoking, BMI, and physical activity were considered as significant predictors of comorbidity among hypertensive patients. Hypertensive patients aged 55 years or older were found to be four times more likely to have comorbidities compared to those aged 18–34 years (AOR = 4.42; 95% CI: 2.65, 7.68). Urban residents with hypertension were approximately seven times more likely to develop comorbidities than their rural counterparts (AOR = 6.67; 95% CI: 2.73, 8.72).

The likelihood of comorbidities was 3.5 times higher among hypertensive patients who consumed alcohol compared to those who did not (AOR = 3.50; 95% CI: 2.94, 11.31). Similarly, hypertensive patients who smoked cigarettes had nearly five times the odds of developing comorbidities compared to nonsmokers (AOR = 4.87; 95% CI: 1.23, 6.53).

In terms of BMI, patients with hypertension with a BMI of 25 kg/m^2^ or higher were 4.55 times more likely to develop comorbidities compared to individuals with a BMI below 18.5 kg/m^2^ (AOR = 4.55; 95% CI: 2.78, 10.91). Moreover, physical activity was found to be inversely associated with comorbidities among hypertensive patients. Those who engaged in physical activity were 48% less likely to have comorbidities compared to physically inactive individuals (AOR = 0.52; 95% CI: 0.24, 0.89) (Table 4).

**Table 4:** Regression analysis of factors associated with comorbidity among patients with hypertension admitted at Dilla University Teaching Hospital, Ethiopia Nov, 30, 2022, to Jan 30, 2023 (*n* = 190).

**Variables**	**Presence of comorbidities**		**COR (95% CI)**	**AOR (95% CI)**
	**Yes**	**No**		
Gender of the participants				
Male	66 (68.04%)	31 (31.96%)	2.59 (1.43, 4.67)	1.36 (0.75, 2.45)
Female	42 (45.16%)	51 (54.84%)	1	
Age of the participants				
18–34	9 (34.62%)	17 (65.38%)	1	1
35–54	47 (54.65%)	39 (45.35%)	2.28 (0.91, 5.67)	2.15 (0.72, 6.06)
≥ 55	52 (66.67%)	26 (33.33%)	3.78 (1.48, 9.62)	4.42 (2.65, 7.68) ^∗^
Marital status				
Single	14 (38.89%)	22 (61.11%)	1	
Married	52 (53.60%)	45 (46.40%)	1.82 (0.83, 3.96)	
Divorced	8 (61.54%)	5 (38.46%)	2.51 (0.68, 9.25)	
Widowed	34 (77.27%)	10 (22.73%)	5.34 (2.02, 14.13)	
Residents				
Rural	33 (34.37%)	63 (65.63%)	1	
Urban	75 (79.79%)	19 (20.21%)	7.54 (3.91, 14.53)	6.67 (2.73, 8.72) ^∗^
Educational status				
Unable to read and write	37 (66.07%)	19 (33.93%)	1	
Grades 1–8 (primary)	38 (56.72%)	29 (43.28%)	0.67 (0.32, 1.40)	
Grades 9–12 (secondary)	24 (50.00%)	24 (50.00%)	0.51 (0.23, 1.13)	
College and above	9 (47.37%)	10 (52.63%)	0.46 (0.16, 1.33)	
Occupational status				
Unemployed	25 (60.97%)	16 (39.03%)	1	
Employed	40 (54.79%)	33 (45.21%)	0.78 (0.36, 1.69)	
Farmers	19 (47.50%)	21 (52.50%)	0.58 (0.24, 1.40)	
Merchants	24 (66.67%)	12 (33.33%)	1.28 (0.50, 3.26)	
Monthly income (in ETB)				
< 1000	27 (35.06%)	50 (64.94%)	1	
1000–3000	51 (80.95%)	12 (19.05%)	7.41 (3.44, 15.95)	2.12 (0.89, 9.89)
> 3000	30 (60.00%)	20 (40.00%)	3.14 (1.50, 6.56)	1.37 (0.34, 3.78)
Alcohol drinking				
Yes	39 (81.25%)	9 (18.75%)	4.58 (2.07, 10.16)	3.50 (2.94, 11.31) ^∗^
No	69 (48.59%)	73 (51.41%)	1	1
Physical activity				
Yes	14 (34.15%)	27 (65.85%)	0.27 (0.13, 0.56)	0.52 (0.24, 0.89) ^∗^
No	94 (63.09%)	55 (36.91%)	1	1
Cigarette smoking				
Yes	28 (75.68%)	9 (24.32%)	2.84 (1.26, 6.42)	4.87 (1.23, 6.53) ^∗^
No	80 (52.30%)	73 (47.70%)	1	1
BMI (kg/m^2^)				
< 18.5	13 (35.14%)	24 (64.86%)	1	
18.5–24.9	72 (59.02%)	50 (40.98%)	2.77 (1.29, 5.93)	1.96 (1.24, 3.57) ^∗^
≥ 25	23 (74.19%)	8 (25.81%)	5.53 (1.94, 15.75)	4.55 (2.78, 10.91) ^∗^
Family history of hypertension				
Yes	48 (55.81%)	38 (44.19%)	0.93 (0.52, 1.65)	
No	60 (57.69%)	44 (42.31%)	1	
Duration with hypertension (in years)				
1–5	42 (41.58%)	59 (58.42%)	1	1
6–10	53 (76.81%)	16 (23.19%)	4.65 (2.35, 9.23)	1.01 (0.98, 7.29)
≥ 11	13 (65%)	7 (35%)	2.61 (0.96, 7.09)	1.46 (0.62, 6.12)

Abbreviations: AOR, adjusted odds ratio; CI, confidence interval; COR, crude odds ratio.

∗ denote significance at *p* value of < 0.05 in the multivariate logistic regression analysis.

## 4. Discussion

Comorbidity in patients with hypertension results in poorer health outcomes, including reduced quality of life and mortality [39, 40]. Assessing comorbidity and associated factors among patients with hypertension is crucial for public health intervention. Therefore, the present study highlights the prevalence of comorbidity and its related factors in patients with hypertension. This study revealed that the prevalence of comorbidities among patients with hypertension was 56.84% (95% CI: 49.73%, 63.68%), consistent with findings from studies conducted in Korea [41], Rome (55%) [42], and Southern Ethiopia (59.7%) [12]. This finding was lower than a similar study conducted in Ethiopia, which reported that 90.8% of hypertensive patients had comorbid conditions [34], and in the United Kingdom, which revealed that two‐thirds of people with hypertension have a comorbidity [43]. The variation in comorbidity prevalence between this study and prior research may stem from differences in the reference population, study methodologies, included disease categories, definitions of comorbidity, and sample size. Moreover, this finding was also higher than that of research conducted in China (35.9%) [16], Korea (22.7%) [44], and the United States (23%) [45]. This disparity could be attributed to differences in sociodemographic variables as well as the growing incidence of NCDs in Africa. Adverse environmental conditions, urbanization, and lifestyle changes in developing countries are contributing to the rise of NCDs [46].

Our data revealed that patients with hypertension aged 55 or older were more likely to develop comorbidities than those aged 18–34 years. This finding was in line with previous research that has shown that the risk of NCDs increases with age [47, 48]. The finding could be attributable to older age, which is a known risk factor for cardiovascular disease, and impaired glucose or diabetes was more common among older patients [49]. Moreover, the lack of physical exercise in this age group may also contribute to the risk of developing comorbidities.

The current study found that patients with hypertension in urban areas were more likely to have comorbidities than those in rural areas [16]. This finding is congruent with a study conducted in China, which found that urban residents had nearly double the prevalence of comorbidities compared to rural areas. The possible reason for this situation is the higher level of comorbidity in hypertensive patients among urban residents, which relates to the prevalence of physical inactivity, unhealthy dietary habits, and higher levels of stress that are known to cause other illnesses such as obesity and cardiovascular disease. Moreover, urban areas are known for being the areas with the highest level of air pollution, noise pollution, and other environmental elements, which also play a major role in aggravating the comorbidities [50, 51]. These factors highlight the necessity for targeted health interventions in urban areas to address lifestyle choices and encourage physical activity. Furthermore, public health policies should be aimed at enhancing access to healthy diets and effective stress management strategies to reduce these comorbidities.

This study revealed that being alcohol‐dependent increases the risk of comorbidities compared to nondrinkers. The finding is consistent with studies conducted in Ethiopia, China, and the United States [10–12]. The association may be due to the direct toxic effect of metabolites, changes in lipid metabolism and glucose metabolism, and hormonal changes that can alter metabolic functions of different organs, leading to the development of other chronic diseases such as heart failure, renal disease, and metabolic syndrome [52]. In addition, extreme levels of drinking can also lead to liver damage, which reduces the body′s ability to process drugs and control blood sugar levels; excessive drinking raises the risk of cardiovascular disease, including heart attack and stroke; and alcohol can interact with antihypertensive medication, thereby reducing their effectiveness or producing side effects. Hence, health providers should assess and treat alcoholism in patients with hypertension to reduce the probability of a comorbid condition.

This study revealed that being a smoker increases the risk of comorbidities compared to being a nonsmoker. This finding was in line with a study conducted in China [53]. Smokers with hypertension are at higher risk for comorbidities; this could be because smoking causes other associated blood rises that lead to an increase in the atherosclerosis process, instigate cardiovascular problems, reduce treatment effectiveness, and initiate systemic inflammation. Thus, smoking cessation is given priority as one of the crucial approaches in the treatment of hypertension, followed by the prevention of the development of additional medical conditions.

In this study, the prevalence of comorbidity among hypertensive patients increased with each subsequent BMI group. Overweight or obesity (BMI ≥ 25 kg/m^2^) was associated with an increased risk of comorbidity among patients with hypertension. The findings might be that overweight or obese patients with hypertension exhibit altered metabolic processes (i.e., insulin resistance), chronic inflammation, and a sedentary lifestyle, which contribute to the development of comorbidity [54]. As a result, the findings highlight the importance of nonpharmacological interventions such as weight loss, physical exercise, and dietary changes.

This study found that physical activity was a protective factor for comorbidities among patients with hypertension, which is in line with a previous study [55]. The possible explanation regarding this phenomenon might be that engaging in physical activity increases glucose uptake by working muscle since exercise increases blood flow to that muscle and eventually proper metabolic function, thereby reducing the occurrence of cardiovascular‐related complications [55, 56]. Furthermore, physical inactivity among patients with hypertension increases the chance of growing comorbidities because it leads to insulin resistance, weight gain, obesity, reduced cardiovascular fitness, metabolic syndrome, and a lack of workout‐precipitated blood pressure control. These factors increase the risk of developing diabetes, cardiovascular disorders, and kidney disease [57]. As part of the hypertension remedy, regular exercise and physical activity need to be promoted to reduce the risk of these troubles and improve standard health results.

The findings of this study have important practical and clinical implications. Understanding comorbidities among hypertensive patients is essential for developing effective management strategies. Routine screening for common comorbidities, alongside patient education on lifestyle risks, is crucial for early detection and improved outcomes. Integrated, multidisciplinary care models are necessary for coordinated management, enhancing patient engagement and adherence to treatment plans. By improving communication among healthcare providers, these approaches can optimize healthcare resources and improve clinical outcomes, particularly in resource‐limited settings. Prioritizing these strategies could significantly reduce the burden of hypertension and promote overall public health while also guiding future research into comorbidities in this population.

### 4.1. Strength and Limitation of the Study

The strength of this study is the use of both structured interviews and a review of medical records to collect data, while conducting the study in selected healthcare facilities in Southern Ethiopia ensures that these findings are relevant for the local context, to help inform targeted interventions and healthcare policies. There are certain limitations to this study. Due to a lack of practical methods for assessing other NCDs, we only identified a few comorbid disorders and were also unable to identify comorbidity with communicable diseases (e.g., HIV/AIDS), which may have underestimated the burden of comorbidity among patients with hypertension in the research area. Because a cross‐sectional design does not establish temporal relationships between explanatory variables and outcome variables, the observed associations may not be causal. Moreover, as the data was limited to a single institution, the findings of this study might not reflect the general state of comorbidity in Ethiopia.

## 5. Conclusion

In this study, more than half of hypertensive patients had at least one comorbid condition. Patients aged 55 years and older, those residing in urban areas, and individuals who consume alcohol, smoke cigarettes, and have a body mass index (BMI) of 25 kg/m^2^ or higher are more likely to experience comorbidity alongside hypertension. Additionally, engaging in physical activity can reduce the likelihood of developing comorbidities among patients with hypertension. Therefore, we recommend that the hospital provide comprehensive care to address all comorbidities, including hypertension, in order to improve the treatment outcome and prognosis of these disorders. To minimize hypertension‐related comorbidities, encourage healthy lifestyle changes such as maintaining a balanced diet, engaging in regular physical activity, and managing weight effectively. Conduct routine health screenings, educate individuals on the risks associated with alcohol consumption and smoking, and create community programs that promote physical activity. Establish support groups for those managing hypertension and related conditions to exchange experiences and coping strategies. Additionally, enhance healthcare access to ensure early diagnosis and effective management, particularly for older adults. Moreover, future investigations are needed that will address both communicable and noncommunicable disease comorbidities among patients with hypertension.

**Nomenclature**
BMI: body mass index
DBP: diastolic blood pressure
CKD: chronic kidney disease
DM: diabetes mellitus
ETB: Ethiopian birr
HF: heart failure
HTN: hypertension
IRB: institutional review board
LMICs: low‐ and middle‐income countries
NCDs: noncommunicable diseases
SBP: systolic blood pressure
WHO: World Health Organization

## Consent

The authors have nothing to report.

## Disclosure

All authors have read and approved the final manuscript.

## Conflicts of Interest

The authors declare no conflicts of interest.

## Author Contributions

H.E.H., B.A.G., and D.S. conceived and implemented the study, contributed in manuscript development, conducted the analysis, and approved the final version of the document. E.K.T. and M.A.A. contributed to the study′s inception, implementation, analysis, manuscript development, and final approval.

## Funding

No funding was received for this manuscript.

## Supporting Information

Additional supporting information can be found online in the Supporting Information section.

## Supporting information


**Supporting Information 1** File S1. Questionnaires were used to investigate the comorbidity and associated factors among patients with hypertension in the health facilities of Southern Ethiopia.

**Supporting Information 2** File S2. Dataset (Excel format).

## Data Availability

All relevant data are within the manuscript and its supporting information files.
