# Supplementary material for: Prevalence of Comorbidity and Associated Factors Among Patients With Hypertension in Health Facilities of Southern Ethiopia
Source: Biomed Res Int. 2026 Jan 21;2026:7739774. doi: 10.1155/bmri/7739774 (PMC12820984; doi:10.1155/bmri/7739774)
Supplement: Supplementary file 1 — Supporting Information 1 File S1. Questionnaires were used to investigate the comorbidity and associated factors among patients with hypertension in the health facilities of Southern Ethiopia. [file BMRI-2026-7739774-s001.docx]

# **Annex’s**

Annex I: Information Sheet

Dilla University College of Medicine and Health Sciences, school of medicine, graduate studies.

Title of the project: comorbidities and associated factors in hypertensive patients at Dilla University General Hospital, Ethiopia, 2022.

The names of the investigators are Banchayehu Azmeraw, Daniel Sisay, Habtamu Endashaw, Mequanint Ayehu, and Endashaw Kefyalew Temesgen.

Name of the organization: Dilla University

Introduction: This information sheet is prepared for the administrative office of Dilla University General Hospital and aims to verify the purpose of the study, the method and procedure of data collection, the standard of research considering ethical issues, and to get participants consent to conduct this study.

Purpose of the study: To assess the prevalence of comorbidities and associated factors among hypertension in Dilla University General Hospital.

Benefits and risks of the study

Benefits: This research may have no direct benefit for patients who are participating in this study currently, but it mostly has a benefit for care planners, managers, the hospital community, and clients in the future if the concerned body implements tasks recommended by the researcher based on the findings.

Risks: Individual patients will not be subjected to any harm.

Confidentiality: All information collected from this research was kept confidential, will not be revealed to anyone except for the investigator, and is kept in a computer password. Its confidentiality was reassured by collecting information without the name of the client.

Annex 2. Written consent form

Dear participants my name is ______________________. I am here to collect data on behalf of the investigators, who are lecturers at Dilla University College of Medicine and Health Science, Department of Medicine, who want to conduct research on comorbidities and associated factors among hypertension for the partial fulfillment of a degree of medical doctorate for one of the investigators named Banchayehu Azmeraw Gedamu2. I am going to ask some questions about your sociodemographic characteristics, behavioral factors, health-related conditions, and comorbidity status, and measure your weight and height. Your honest response regarding the given questions will provide a valid result for comorbidities and their associated factors, which makes it important to take appropriate intervention; hence, I request that you participate honestly.

Participant Rights: Your participation was entirely based on your voluntary choice and was up to you to decide. There was no penalty if you did not agree to participate. In addition, you have the right not to answer any questions you do not want to. Your decision will not affect your right to get a health service.

Finally, all the information you provided was kept confidential. Only a number identifies your response to questions, never by name. If you agree, you are invited to answer questions. If yes, let us continue.

Annex III. English version questionnaire

Part: 1. Socio-demographic information

Table 1. Questionnaires on sociodemographic characteristic hypertensive patients at Dilla general hospital, Ethiopia, 2022.

| Ser. No | Variables | Response | |
| --- | --- | --- | --- |
| 101 | Sex | 1. Male 2. Female | |
| 102 | Age (in year) | ----------------- | |
| 103 | Residence | 1. Urban 2. Rural | |
| 104 | Marital status | 1. Single 2. Married  3. Widowed 4. Divorced | |
| 105 | Educational status | 1. Unable to read and write 2. Primary school ( grade 1-8) 3. Secondary school (grade 9-12) 4. College and above | |
| 106 | Monthly income (in ETB) | ---------------- | |
| 107 | Occupation | | 1. unemployed 2. government employee 3. private employee 4. Farmer 5. Merchant |

Part: 2. Behavioral factors

Table 2.Questionnaires on behavioral factors among hypertensive patients at Dilla general hospital, southern Ethiopia, 2022

| 201 | Did you smoke a cigarette or cigar, even just one puff, in the past 28 days? | 1. Yes 2. No | |
| --- | --- | --- | --- |
| 203 | Did you drink alcohol ((bottle of regular beer (300 ml), local alcohol "Areki" (30 ml), or one medium-sized glass of wine (120 ml) alcohol) in the past 28 days? | | 1. Yes 2. No |
| 205 | Did you chew chat in the past 28 days at least once per day?? | | 1.Yes  2.No |
| 207 | Do you engage in physical exercise | | 1.yes 2, no |
| 208 | If yes to question 207, how many times per week? | | _______________ in number |

Part: 3. Clinical factors and comorbidity status

Table 3. Questionnaire on Clinical and comorbidity status of hypertensive patients at Dilla general hospital, southern Ethiopia, 2022

| 301. Is there a family member with a known history of HTN? | 1.yes  2.No |
| --- | --- |
| 302. How long was it after your hypertension diagnosis? (Years) | ----------------------- |
| 303. Are you with any antihypertensive drug?  ( cross-check with medical records) | 1. Yes 2. No |
| 304. For how long do you take this drug? | __________________ . |
| Comorbidities Question |  |
| 305. Have you ever been diagnosed by a health care professional as having the following chronic conditions?" The responses included diabetes mellitus, heart failure, dyslipidemia, stroke, and chronic renal illness? (Verify the comorbidity information with the patient's registration books and medical records) | 1. Yes 2. No |
| 305.1. Have you ever diagnosed with DM | 1. Yes 2. No |
| 305.2 .Chronic renal failure | 1. Yes 2. No |
| 305.3 .have you ever diagnosed with stroke | 1. Yes 2. No |
| 305.4. Have you diagnosed for heart failure | 1. Yes 2. No |
| 305.5 .Dyslipidemias | 1. Yes  2. No |

Part 4. Physical measurements

Table 4. Physical measurements of**,** hypertensive patients at Dilla general hospital, Ethiopia, 2022

| Blood pressure; Systolic ______mmHg, Diastolic: ________mmHg (To find out if they have hypertension, cross-check their medical data) | |
| --- | --- |
|  |  |
|  |  |
| Measure the Height of the participants ( in cm)__________________ | |
| Measure the weight of the participants ( in kg)__________________ | |
| BMI ( $\frac{\mathrm{kg}}{{cm}^{2}}$ ) |  |
